# Early Botulinum Toxin Type A Injection May Improve Motor Recovery in Patients with Post-Stroke Spasticity: A Secondary Analysis from a Longitudinal Cohort Study

**DOI:** 10.3390/toxins17110558

**Published:** 2025-11-13

**Authors:** Alessandro Picelli, Andrea Santamato, Michela Cosma, Alessio Baricich, Carmelo Chisari, Marzia Millevolte, Cristina Del Prete, Ilenia Mazzù, Rita Di Censo, Nicola Smania, Mirko Filippetti

**Affiliations:** 1 Section of Physical and Rehabilitation Medicine, Department of Neurosciences, Biomedicine and Movement Sciences, University of Verona, 37134 Verona, Italynicola.smania@univr.it (N.S.); mirko.filippetti@univr.it (M.F.); 2 Spasticity and Movement Disorders “ReSTaRt”, Physical Medicine and Rehabilitation Section, Department of Medical and Surgical Sciences, University of Foggia, 71122 Foggia, Italy; andrea.santamato@unifg.it; 3 Neuroscience and Rehabilitation Department, Ferrara University Hospital, 44124 Ferrara, Italy; m.cosma@ospfe.it; 4 Department of Biomedical Sciences, Humanitas University, 20072 Milan, Italy; alessio.baricich@hunimed.eu; 5 Section of Neurorehabilitation, Department of Translational Research and New Technologies in Medicine and Surgery, University of Pisa, 56126 Pisa, Italy; carmelo.chisari@unipi.it; 6 Neurorehabilitation Unit, Ancona University Hospital, 60123 Ancona, Italy; marzia.millevolte@ospedaliriuniti.marche.it; 7 Department of Physical and Rehabilitation Medicine, ASL Lecce, 73100 Lecce, Italy; dipriab@asl.lecce.it; 8 IRCCS Santa Lucia Foundation, 00179 Rome, Italy; i.mazzu@hsantalucia.it

**Keywords:** botulinum toxins, muscle spasticity, rehabilitation, therapeutics

## Abstract

Spasticity after stroke impairs motor control, delays recovery, and reduces quality of life. Botulinum toxin type A is the first-line treatment, but it is often administered in the chronic phase, potentially limiting its impact on rehabilitation. Emerging evidence suggests that earlier treatment may enhance recovery, though functional benefits remain uncertain. We conducted a secondary analysis of a multicenter, open-label, longitudinal cohort study to investigate whether the timing of the first botulinum toxin type A injection influences outcomes in post-stroke patients naïve to this treatment. All participants received botulinum toxin injections combined with conventional rehabilitation. Assessments were performed at baseline and at 4, 12, and 24 weeks post-injection. The primary outcome was muscle tone; secondary outcomes included motor strength, sensorimotor recovery, and global disability. Statistical analyses used mixed-effects models and trend tests. Patients treated within 90 days of stroke onset showed greater reductions in spasticity at 4 and 12 weeks compared with later treatment. Despite having more severe baseline impairments, early treated patients demonstrated faster and more pronounced improvements in upper-limb strength, sensorimotor recovery, and global disability. Early toxin administration is associated with enhanced reduction in spasticity and improved motor recovery, particularly in patients with severe initial deficits.

## 1. Introduction

Post-stroke spasticity (PSS) has a reported prevalence ranging from 4–27% within the first 6 weeks after stroke onset, 19% at 3 months, 21.7–42.6% between 4 and 6 months, and 17–38% at 12 months [1,2,3]. According to Li, Francisco and Rymer [4], PSS manifests “as velocity- and muscle length–dependent increase in resistance to externally imposed muscle stretch. It results from hyperexcitable descending excitatory brainstem pathways and from the resultant exaggerated stretch reflex responses. Other related motor impairments, including abnormal synergies, inappropriate muscle activation, and anomalous muscle coactivation, coexist with spasticity and share similar pathophysiological origins.”

Spasticity can significantly limit voluntary motor control, delay functional recovery, and negatively affect quality of life in stroke survivors [5,6]. Timely identification and management of PSS are therefore essential to optimize rehabilitation outcomes and to prevent long-term sequelae such as pain, joint deformities, and fixed contractures [6].

Botulinum toxin type A (BoNT/A) is currently regarded as the first-line pharmacological treatment for PSS. Its efficacy in reducing muscle tone and improving passive function has been well documented for both upper and lower limbs [7]. Nevertheless, in everyday clinical practice, BoNT/A is frequently administered during the chronic phase, several months after stroke onset, potentially limiting its effects on motor recovery and functional independence [1,8,9].

Emerging evidence suggests that earlier administration of BoNT/A (i.e., during the subacute phase post-stroke) may amplify the benefits of rehabilitation by attenuating abnormal muscle tone before it becomes established and by facilitating more effective motor relearning [10,11,12]. However, the optimal timing for initiating BoNT/A treatment remains controversial, and only a limited number of studies have systematically investigated its impact on functional outcomes when administered early [10,11,13,14,15,16].

In a previous multicenter, longitudinal cohort study, Picelli and colleagues evaluated the effects of BoNT/A in a real-world population of treatment-naïve patients with PSS who received injections within 12 months of stroke onset [17]. The primary outcome was the Modified Ashworth Scale (MAS) [18], which showed a significant reduction in muscle tone at both 4 and 12 weeks post-injection, particularly in patients treated within 90 days of stroke. However, the potential association of early BoNT/A treatment with motor recovery (beyond tone reduction) was not specifically addressed in that analysis [17].

The present secondary analysis aims to fill this gap by evaluating whether the timing of the first BoNT/A injection after stroke onset is associated with motor outcomes in patients with PSS in routine clinical settings. Leveraging data from the previously conducted multicenter cohort study [17], this analysis seeks to provide a deeper understanding of the motor impact of early spasticity management. The findings may contribute to refining clinical decision-making regarding the optimal timing of BoNT/A intervention in stroke rehabilitation, ultimately aiming to improve recovery trajectories and long-term outcomes for patients with PSS.

## 2. Results

A total of 83 patients with PSS were included in the study. The mean age at stroke onset was 63.9 ± 12.5 years, and 62.7% of the participants were male. The average time between stroke onset and the first BoNT/A injection was 136.1 ± 95.1 days. Most strokes were ischemic in origin (69.9%), and the lesion was more commonly located in the right hemisphere (54.2%). BoNT/A injections were administered in different clinical settings: 48.2% of patients were treated as inpatients, 39.8% as outpatients, and 12.0% within day-hospital programs.

Baseline characteristics stratified by the time elapsed between stroke onset and botulinum toxin injection are reported in Table 1.

### 2.1. BoNT/A Dosing, Muscle Groups and Injection Procedures

The total injected dose per treatment was individualized according to the Italian label, clinical presentation and target muscles. Across the cohort, the median (IQR) doses by formulation were AbobotulinumtoxinA 1000 U (700–1075), IncobotulinumtoxinA 300 U (212.5–400) and OnabotulinumtoxinA 300 U (250–430). Within each formulation, there were no statistically significant differences in total dose between the early (≤90 days from stroke onset) and late (>90 days) groups (AbobotulinumtoxinA: 900 U vs. 1000 U, *p* = 0.138; IncobotulinumtoxinA: 275 U vs. 350 U, *p* = 0.359; OnabotulinumtoxinA: 265 U vs. 300 U, *p* = 0.066).

Details about the injected muscle groups are reported in Table 2.

The most commonly used injection technique was ultrasound-guided (65.1%), followed by manual needle placement (21.7%) and electrical stimulation (13.3%). The most frequently adopted dilution was 2 mL per vial (67.5%).

### 2.2. Muscle Spasticity

A total of 1167 MAS measurements were recorded across various muscle groups. MAS scores showed a significant reduction in muscle tone at 4 and 12 weeks post-injection compared to baseline. Although the reduction persisted at 24 weeks, it was less pronounced. The cumulative logistic mixed-effects model confirmed a significant decrease in MAS scores at 4 weeks (OR = 0.05; 95% CI: 0.03–0.09; *p* < 0.001), 12 weeks (OR = 0.06; 95% CI: 0.04–0.11; *p* < 0.001), and 24 weeks (OR = 0.24; 95% CI: 0.14–0.41; *p* < 0.001), relative to baseline. A significant interaction was found between follow-up time and the timing of BoNT/A administration. Patients treated within 90 days from stroke onset had significantly lower MAS scores at both 4 weeks (OR = 2.05; *p* = 0.044) and 12 weeks (OR = 2.87; *p* = 0.002) compared to those treated later. Multivariate analysis identified age > 70 years (OR = 0.35; 95% CI: 0.15–0.80; *p* = 0.013) and higher vial dilution (OR = 0.12 per 1 mL increase; 95% CI: 0.05–0.30; *p* < 0.001) as independent predictors of greater MAS score reduction. No significant interaction was observed between the treatment setting (inpatient vs. outpatient) and MAS outcomes (*p* = 0.29).

### 2.3. Sensorimotor Recovery and Disability

The Kendall trend test revealed a statistically significant upward trend in Motricity Index (MI) scores over time for both the lower limb (*p* = 0.002) and the upper limb (*p* = 0.046), indicating progressive motor recovery across the cohort. At baseline, patients who received BoNT/A within 90 days of stroke onset (early treatment group) exhibited lower MI scores compared to those treated after 90 days (late treatment group), suggesting greater initial motor impairment. However, descriptive analyses indicated that the early treatment group showed a steeper improvement trajectory, particularly in the upper limb. To further examine these trends, a linear mixed-effects model was applied to MI scores, incorporating fixed effects for time, treatment group (early vs. late), and their interaction, while accounting for repeated measures within subjects. The model confirmed a significant main effect of time (*p* < 0.01), indicating consistent improvement in MI scores across all follow-up assessments. Patients in the late treatment group demonstrated higher overall MI scores (coefficient = +13.17, *p* = 0.027), consistent with their better baseline motor function. Although the interaction terms between time and treatment group did not reach statistical significance, they suggested a trend toward faster motor recovery in the early treatment group. Table 3 provides an overview of baseline and follow-up descriptive statistics (mean and SD) for the MI.

Table 4 presents baseline and follow-up descriptive statistics (mean and SD) for the MI, stratified by early (≤90 days post-stroke) and late (>90 days) treatment groups.

Across the 24-week follow-up, Fugl-Meyer Assessment (FMA) scores demonstrated a consistent and statistically significant improvement in both limbs, with more pronounced gains observed in the lower limb. For the upper limb, significant improvements were observed in total motor function (*p* = 0.016), speed and coordination (*p* = 0.045), and pain reduction (*p* = 0.007). Although improvements in wrist and hand subcomponents were noted, they did not reach statistical significance individually. For the lower limb, the analysis revealed significant gains in total motor function (*p* < 0.001), speed and coordination (*p* < 0.001), and sensation (*p* = 0.049), while passive mobility and pain domains remained stable over time. Table 5 provides an overview of baseline and follow-up descriptive statistics (mean and SD) for the FMA.

To further explore the effect of treatment timing, a linear mixed-effects model was applied to FMA scores, incorporating fixed effects for time, treatment group (early vs. late), and their interaction. The model confirmed a strong main effect of time (*p* < 0.001), indicating progressive functional recovery in the overall cohort. Importantly, a significant interaction between time and treatment group was observed at 12 weeks (*p* = 0.004), suggesting that patients treated within 90 days of stroke onset experienced a more rapid improvement in FMA scores compared to those treated later. Descriptive analysis also showed that patients in the early treatment group started from a lower baseline FMA score, particularly in the upper limb, but demonstrated a steeper recovery trajectory over time. This pattern supports the hypothesis that early BoNT/A administration may facilitate motor relearning and enhance responsiveness to rehabilitation, especially in patients with more severe initial impairments.

Table 6 presents baseline and follow-up descriptive statistics (mean and SD) for the FMA of the upper and lower extremities, stratified by early (≤90 days post-stroke) and late (>90 days) treatment groups.

Global disability, assessed using the Modified Rankin Scale (MRS), showed a gradual decrease over the 24-week follow-up period, indicating a significant improvement (*p* = 0.039). The mixed-effects model confirmed a significant effect of time (baseline coefficient = +0.35; *p* < 0.001), though no significant between-group differences or interaction effects were detected.

**Figure 1:**
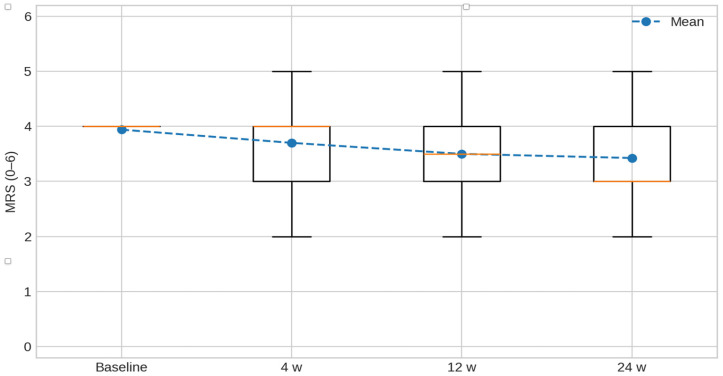
Modified Rankin Scale (MRS, 0–6) over time. Box-and-whisker plots depict the distribution of MRS at baseline, 4, 12, and 24 weeks; boxes show the median and interquartile range, whiskers indicate the range, and the mean is overlaid with a line marker. Lower scores indicate less disability.

MRS values over time are shown in Figure 1. The distribution at each assessment (baseline, 4, 12, and 24 weeks) is presented as box-and-whisker plots (median and interquartile range; whiskers indicate the range; the mean is overlaid with a line marker), illustrating the progressive decrease in disability across follow-up.

## 3. Discussion

This secondary analysis from a longitudinal cohort study [17] provides new insights into the clinical impact of early versus delayed administration of BoNT/A in treatment-naïve patients suffering from PSS, with a particular focus on motor function, sensorimotor recovery, and global disability. Through a combination of non-parametric group comparisons and mixed-effects modeling, we observed that early BoNT/A treatment (≤90 days from stroke onset) was associated with more favorable outcomes in terms of muscle tone reduction and motor recovery, particularly in the upper limb. These findings align with and expand upon current literature that increasingly supports early BoNT/A administration as a potentially disease-modifying intervention in the subacute phase of stroke.

### 3.1. Clinical Implications for Sensorimotor Recovery

The MI, a validated measure of voluntary motor strength, showed progressive improvement over time in both early and late treatment groups. However, patients who received BoNT/A within 90 days post-stroke exhibited a steeper trajectory of improvement, especially in the upper limb. While between-group differences did not reach statistical significance in Mann–Whitney tests at individual timepoints, the trend favored early intervention. Clinically, these findings are consistent with those reported in the primary analysis of this cohort [17] and support the hypothesis that early BoNT/A administration may not only reduce spasticity but also create a more favorable neuromuscular environment for motor relearning. This is particularly relevant during the early subacute phase, when neuroplasticity is maximal and rehabilitation is most effective. The observed trend toward faster MI improvements in early treated patients supports the idea that BoNT/A may act as a facilitator of motor recovery when administered within the critical window of neural reorganization. Similarly, the ONTIME study [19] demonstrated that early BoNT/A treatment delayed the need for reinjection and was associated with sustained reductions in muscle tone, indirectly supporting enhanced motor recovery potential.

The FMA is a comprehensive tool for evaluating sensorimotor recovery after stroke. In our analysis, the FMA scores for the upper limb showed a significant difference between early and late treatment groups at baseline (*p* = 0.026), with the early group starting from a lower functional level. Despite this, the early group demonstrated a comparable or greater rate of improvement over time, as confirmed by the mixed-effects model. These findings together with those reported by the main analysis of this study published in 2021, suggest a potential for early BoNT-A to accelerate functional recovery [17]. The meta-analysis by Rosales and collaborators [10] also supports this interpretation, showing that early BoNT-A treatment (within 3 months post-stroke) significantly reduced hypertonia, although effects on disability and function were less consistent. However, the authors acknowledged that most included studies were underpowered to detect functional changes and that longer follow-up periods might be necessary to observe meaningful improvements. Our findings contribute to this discussion by showing that early BoNT-A treatment may help bridge the functional gap in more severely affected patients, potentially by reducing spastic interference and allowing more effective participation in task-specific training. This has important implications for rehabilitation planning, as it suggests that early identification and treatment of spasticity could enhance the therapy responsiveness and long-term functional outcomes.

### 3.2. Global Disability and Participation

Global disability, assessed using the MRS, improved progressively in both treatment groups over the 24-week period. Although between-group differences at individual timepoints were not statistically significant, the mixed-effects model indicated a more favorable trajectory in the early treatment group, particularly in interaction effects. These results are consistent with findings from Lindsay et al. [11], who also reported MRS improvement over time following BoNT/A treatment. Although MRS was not their primary outcome, their observations support the hypothesis that early spasticity management may contribute to enhanced overall functional status and social participation. In addition, a recent economic analysis by the same Authors [20] showed that early BoNT/A treatment was associated with lower contracture-related costs and a more favorable cost-effectiveness profile, as reflected by improvements in Barthel Index and Action Research Arm Test scores. These findings suggest that early intervention may not only yield clinical benefits but also reduce the long-term burden on healthcare systems and caregivers. From a clinical perspective, the improvements observed in MRS scores further support the integration of BoNT/A into early-stage multidisciplinary stroke rehabilitation, promoting independence, reducing the need for long-term assistance, and enhancing quality of life.

### 3.3. Limitations

Despite the promising findings, several limitations must be acknowledged. First, the study design was observational and non-randomized, which introduces the possibility of selection bias and limits the ability to establish causal relationships. Although we attempted to control for confounding variables through statistical modeling, unmeasured factors such as stroke severity, lesion location, and rehabilitation intensity may have influenced the results. Second, the sample size was relatively small, particularly when stratified by treatment timing and follow-up intervals. This may have limited the statistical power to detect significant differences between groups, especially in the presence of inter-individual variability in recovery trajectories. Third, the assessment of functional outcomes was limited to standardized clinical scales (MI, FMA, MRS), which, while validated, may not fully capture patient-centered outcomes such as quality of life, goal attainment, or satisfaction with treatment. Furthermore, although several outcomes showed statistically significant improvements over time, none of these changes exceeded established Minimal Detectable Change (MDC) or Minimal Clinically Important Difference (MCID) thresholds. This indicates that the observed effects might not translate into clinically meaningful benefits. Therefore, the results should be interpreted with caution, and future studies should incorporate MDC and MCID benchmarks as predefined criteria to better evaluate the practical impact of early BoNT/A administration. Fourth, the timing and dosage of BoNT/A injections were not standardized across centers, reflecting real-world practice but potentially introducing variability in treatment effects. Similarly, the type and intensity of concomitant rehabilitation interventions were not controlled, which may have influenced the observed outcomes. Fifth, the follow-up period was limited to 24 weeks, which may not be sufficient to capture the long-term effects of early BoNT/A treatment on functional recovery, reinjection intervals, or prevention of contractures. Longer-term studies are needed to determine whether the early benefits observed in this study translate into sustained improvements in independence and participation. Finally, it is important to acknowledge that the observed improvements in motor function over time may partly reflect the natural course of stroke recovery rather than a direct effect of BoNT/A. Although our mixed-effects models indicated an interaction between treatment timing and recovery trajectories, the observational design of this study precludes causal inference. Unmeasured factors such as rehabilitation intensity, lesion characteristics, and spontaneous neuroplasticity could have influenced outcomes. Therefore, these findings should be interpreted as associative rather than confirmatory, and randomized controlled trials are needed to determine whether early BoNT/A administration provides benefits beyond usual recovery.

## 4. Conclusions

This secondary analysis suggests that early administration of BoNT/A (ideally within 90 days of stroke onset) may be associated with enhanced motor recovery and additional functional gains in patients with post-stroke spasticity. These findings support the integration of BoNT/A into early-stage multidisciplinary rehabilitation programs, particularly for individuals with severe initial motor deficits. Clinicians should consider early identification and treatment of spasticity to optimize responsiveness to therapy and potentially improve long-term outcomes. However, given the observational nature of the study, randomized controlled trials are needed to confirm these associations and to evaluate the sustained impact of early BoNT/A treatment on disability, reinjection intervals, and participation in rehabilitation.

## 5. Materials and Methods

### 5.1. Study Design and Setting

This study is a secondary analysis of data obtained from a multicenter, open-label, longitudinal cohort study conducted between June 2015 and December 2018 in nine university and clinical hospitals across Italy [17]. The study adhered to the principles of the Declaration of Helsinki and was approved by the local ethics committee (Comitato Etico per la Sperimentazione Clinica delle Province di Verona e Rovigo, protocol code 392CESC). All participants provided written informed consent prior to enrollment. The study was registered at ClinicalTrials.gov (NCT04404868). This secondary analysis follows the STROBE guidelines for reporting observational studies.

### 5.2. Participants

Eligible participants were adults (≥18 years) with a first-ever unilateral ischemic or hemorrhagic stroke confirmed by neuroimaging, and a time from stroke onset of less than 12 months. Inclusion criteria required the presence of clinically significant spasticity, defined as a score of at least 1+/4 on the MAS in at least one muscle group of the affected limb [18], and preserved voluntary movement of the antagonist muscles, defined as a score ≥ 2/5 on the Medical Research Council scale [21]. Only BoNT/A-naïve patients were included. Concomitant use of other antispastic medications (e.g., oral muscle relaxants) was not allowed during the study. Exclusion criteria included: participation in other clinical trials, fixed contractures (MAS = 4/4), bony deformities, prior surgical or neurolytic procedures for spasticity, and any other neurological or orthopedic condition affecting the limb under evaluation.

### 5.3. Intervention

All patients received BoNT/A injections as part of routine clinical care. The specific formulation (AbobotulinumtoxinA, OnabotulinumtoxinA, or IncobotulinumtoxinA), dosage, dilution, and injection technique (ultrasound guidance, electrical stimulation, or manual needle placement) were determined by the treating physician, in line with local protocols and clinical guidelines [22,23]. BoNT/A was administered to upper and/or lower limb muscles based on individual spasticity patterns, including shoulder adduction/internal rotation, elbow flexion, wrist and finger flexion, thumb-in-palm deformity, hip adduction, knee flexion or extension, and equinovarus foot.

All participants received conventional rehabilitation therapy as per local clinical practice, including physical therapy, occupational therapy, and functional training. Even if the intensity, duration, and setting of rehabilitation (inpatient, outpatient, or day hospital) were defined according to the current guidelines [24,25], they varied among centers and were not standardized, reflecting real-world clinical conditions.

### 5.4. Outcome Measures

Patients were assessed at baseline before BoNT/A injection (T0), and at 4 weeks (T1), 12 weeks (T2), and 24 weeks (T3) post-injection. The primary outcome measure was the MAS, a 6-point ordinal scale used to evaluate resistance to passive movement and muscle tone [18]. Secondary outcomes included MI [26], FMA [27], and MRS [28]. The MI assesses voluntary motor strength in upper and lower limbs, with subtests for shoulder abduction, elbow flexion, and pinch grip (upper limb), and hip flexion, knee extension, and ankle dorsiflexion (lower limb). Scores range from 0 to 100 per limb [26]. The FMA is a comprehensive scale for evaluating post-stroke sensorimotor recovery, comprising domains for motor function (upper limb max = 66; lower limb = 34), sensory function (max = 24), balance (max = 14), joint range of motion (max = 44), and joint pain (max = 44). Total maximum score: 226 [27]. The MRS is a global disability scale ranging from 0 (no symptoms) to 6 (death) [28].

### 5.5. Statistical Analysis

Descriptive statistics were used to summarize demographic and clinical characteristics. Continuous variables were reported as mean ± standard deviation (SD) or median and interquartile range (IQR), depending on distribution. Categorical variables were summarized using frequencies and percentages. Normality of continuous variables was assessed using the Shapiro–Wilk test.

Between-group comparisons were performed using Student’s *t*-test or Wilcoxon rank-sum test for continuous variables, and chi-square or Fisher’s exact test for categorical variables. To assess changes over time, repeated measures of ordinal outcomes were analyzed using the Kendall trend test. The primary analysis examined the effect of time from stroke onset to first BoNT/A injection (≤90 days vs. >90 days) on MAS and secondary functional outcomes.

A cumulative logistic mixed-effects model was employed to evaluate the association between the timing of BoNT/A administration and MAS scores over time. The model accounted for repeated measures within subjects and across muscle groups and included fixed effects for time (T0, T1, T2, T3), age group (<60, 60–70, >70 years), sex, BoNT/A dilution, and time from stroke onset to injection. Random effects were specified for subject-time and muscle group-time interactions. Odds ratios (ORs) with 95% confidence intervals (CIs) were reported. Model selection was guided by the Akaike Information Criterion (AIC), and second-order interactions were tested for significance.

Post hoc pairwise comparisons were performed on estimated marginal means with Bonferroni correction. Tables report both adjusted *p*-values and effect sizes (Hedges’ g) for between-group contrasts.

All statistical analyses were performed using R 4.2.3 for macOS (https://www.r-project.org/; accessed on 6 June 2025), including the ordinal package for cumulative logistic regression. A two-tailed *p*-value < 0.05 was considered statistically significant.

## Figures and Tables

**Table 1:** Baseline characteristics of our sample.

Outcome	<90 Days Mean (SD)	≥90 Days Mean (SD)
Motricity index UL	22.19 (19.29)	34.83 (28.15)
Motricity index LL	39.92 (21.27)	47.37 (22.00)
Fugl-Meyer Assessment UL	10.81 (10.65)	19.35 (16.41)
Fugl-Meyer Assessment UL	14.73 (6.72)	16.04 (7.00)
Modified Rankin scale	4.03 (0.69)	3.87 (0.65)

Abbreviations: SD, Standard Deviation.

**Table 2:** Injected muscles.

Upper Limb Muscles (n)	Lower Limb Mean (n)
Flexor digitorum superficialis (48)	Gastrocnemius medialis (32)
Flexor carpi ulnaris (35)	Gastrocnemius lateralis (30)
Biceps brachii (34)	Soleus (28)
Flexor carpi radialis (34)	Tibialis posterior (14)
Flexor digitorum profundis (32)	Flexor digitorum longus (8)
Pronator teres (27)	Flexor hallucis longus (7)
Brachialis (20)	Hamstrings (4)
Flexor pollicis longus (18)	Rectus femoris (4)
Pectoralis major (18)	Gracilis (3)
Brachioradialis (16)	Tibialis anterior (2)
Subscapularis (3)	Extensor hallucis longus (2)
Interossei (2)	Flexor hallucis brevis (1)
Latissimus dorsi (1)	Flexor digitorum brevis (1)
Opponens pollicis (1)	Peroneus longus (1)

Abbreviations: n, number of injections.

**Table 3:** Motricity Index at each time point.

Time Point	Upper Limb Mean (SD)	Lower Limb Mean (SD)
Baseline	29.19 (25.26)	44.05 (21.87)
4 weeks	33.39 (26.58)	49.59 (19.64)
12 weeks	36.19 (27.57)	52.79 (20.82)
24 weeks	37.86 (27.95)	55.39 (20.59)

Abbreviations: SD, Standard Deviation.

**Table 4:** Motricity Index at each time point stratified by early and late injection.

	≤90 Days Post-Stroke Mean (SD)	>90 Days Post-Stroke Mean (SD)	Post Hoc Comparison *p* Value (Effect Size)
Upper limb			
Baseline	22.3 (19.0)	35.0 (28.5)	0.080 (−0.51)
4 weeks	27.5 (23.9)	38.5 (27.9)	0.110 (−0.42)
12 weeks	31.5 (26.1)	40.3 (28.4)	0.166 (−0.32)
24 weeks	32.9 (26.0)	42.1 (29.1)	0.193 (−0.33)
Lower limb			
Baseline	39.7 (21.0)	47.7 (22.1)	0.294 (−0.36)
4 weeks	45.6 (19.1)	53.0 (19.7)	0.481 (−0.38)
12 weeks	51.2 (22.7)	54.1 (19.3)	0.874 (−0.14)
24 weeks	54.9 (20.9)	55.8 (20.6)	0.912 (−0.04)

Abbreviations: SD, Standard Deviation.

**Table 5:** Fugl-Meyer Assessment at each time point.

Sub-Items	Baseline Mean (SD)	4 Weeks Mean (SD)	12 Weeks Mean (SD)	24 Weeks Mean (SD)
Upper extremity (0–36)	10.9 (8.8)	12.3 (9.2)	14.4 (10.0)	14.9 (10.6)
Wrist (0–10)	1.0 (2.4)	1.7 (2.9)	2.2 (3.0)	2.3 (3.3)
Hand (0–14)	2.6 (3.8)	3.0 (4.2)	3.7 (4.7)	3.8 (4.9)
Coordination/Speed (0–6)	1.6 (1.8)	1.7 (1.8)	2.1 (2.0)	2.2 (2.0)
Motor function (0–66)	15.5 (14.6)	18.6 (16.9)	21.5 (17.8)	22.4 (18.9)
Sensation (0–12)	9.2 (3.1)	9.4 (3.1)	9.5 (3.0)	9.9 (2.9)
Passive joint motion (0–24)	18.3 (4.3)	19.9 (3.6)	19.3 (4.3)	19.0 (4.5)
Joint pain (0–24)	15.7 (7.1)	17.4 (6.4)	18.0 (6.3)	18.1 (6.7)
Lower extremity (0–28)	13.6 (5.8)	15.5 (5.7)	16.2 (6.1)	16.5 (6.2)
Coordination/Speed (0–6)	2.1 (1.7)	2.7 (1.8)	3.0 (1.8)	3.2 (1.8)
Motor function (0–34)	15.4 (6.8)	17.5 (6.6)	18.2 (6.6)	18.9 (6.8)
Sensation (0–12)	9.1 (3.1)	9.8 (2.7)	9.7 (2.9)	10.3 (2.8)
Passive joint motion (0–20)	16.6 (3.2)	17.3 (3.0)	17.3 (3.1)	17.4 (3.0)
Joint pain (0–20)	17.8 (4.5)	17.3 (6.0)	18.4 (4.0)	17.3 (6.0)

Abbreviations: SD, Standard Deviation.

**Table 6:** Fugl-Meyer Assessment scores for the upper and lower extremities, stratified by early and late injection.

Sub-Items	Baseline Mean (SD)	4 Weeks Mean (SD)	12 Weeks Mean (SD)	24 Weeks (Mean SD)
**Upper extremity (0–36)**				
≤90 days post-stroke	8.6 (6.5)	10.9 (7.5)	14.3 (9.6)	14.5 (9.8)
>90 days post-stroke	12.9 (10.1)	13.6 (10.4)	14.5 (10.6)	15.2 (11.3)
**Post hoc comparison** *p* value (effect size)	0.026 (−0.49)	0.221(−0.29)	0.283 (−0.02)	0.212 (−0.07)
**Lower extremity (0–28)**				
≤90 days post-stroke	13.1 (5.4)	15.6 (5.8)	16.7 (6.2)	17.1 (6.1)
>90 days post-stroke	14.2 (6.2)	15.4 (5.7)	15.8 (6.0)	16.0 (6.2)
**Post hoc comparison** *p* value (effect size)	0.322 (−0.18)	0.771 (0.04)	0.664 (0.15)	0.439 (0.16)

Abbreviations: SD, Standard Deviation.

## Data Availability

Dataset available on request from the authors. This is a secondary analysis of data. The information was provided as it is consistent with that reported in published article showing primary analysis of the data (Picelli et al. Early Botulinum Toxin Type A Injection for Post-Stroke Spasticity: A Longitudinal Cohort Study. Toxins (Basel). 2021 May 24;13(6):374) [17].

## Abbreviations

The following abbreviations are used in this manuscript:

PSS	Post-stroke spasticity
BoNT/A	Botulinum toxin type A
MAS	Modified Ashworth scale
OR	Odds ratio
CI	Confidence interval
MI	Motricity index
SD	Standard deviation
FMA	Fugl-Meyer assessment
MRS	Modified Rankin scale
MDC	Minimal detectable change
MCID	Minimal clinically important difference
CT	Computerized tomography
MRI	Magnetic resonance imaging
IQR	Interquartile range
